# Influence of ZnO nanoparticles on *Candida albicans* isolates biofilm formed on the urinary catheter

**Published:** 2018-12

**Authors:** Seyedeh Sedigheh Hosseini, Ezzatollah Ghaemi, Faramarz Koohsar

**Affiliations:** 1 Laboratory Sciences Research Center, Golestan University of Medical Sciences, Gorgan, Iran; 2 Department of Laboratory Sciences, Faculty of Paramedicine, Golestan University of Medical Sciences, Gorgan, Iran; 3 Department of Microbiology, Faculty of Medicine, Golestan University of Medical Sciences, Gorgan, Iran; 4 Department of Medical Parasitology and Mycology, School of Public Health, Tehran University of Medical Sciences, Tehran, Iran

**Keywords:** Urinary tract infection, ZnO nanoparticle, Biofilm, *Candid albicans*, Catheter

## Abstract

**Background and Objectives:**

The aim of this study was to determine the effect of zinc oxide nanoparticle (ZnO-np) solution in the surface catheter on *C. albicans* adhesion and biofilm formation.

**Materials and Methods:**

Out of 260 isolates from urinary catheter, 133 were determined as *C. albicans* by common phenotypic and genotyping methods. ZnO nanoparticles with 30 nm were made by the sol-gel method, which was confirmed by XRD (X-ray diffraction) and scanning electron microscope (SEM) methods. Candidal adhesion and biofilm assays were performed on catheter surfaces for 2 and 48 hours, respectively. The effect of sub-MIC (minimum inhibitory concentrations) and MIC concentrations of ZnO-np on biofilm formation was evaluated after 24 hours using Crystal violet (CV), colony-forming unit (CFU), and SEM.

**Results:**

Out of 133 *C. albicans* isolates, 20 (15%) fluconazole-resistant and 113 (85%) susceptible isolates were determined by the disk diffusion method. Results showed that both isolates adhered to biofilm formation on the catheter surfaces. A significantly (P< 0.05) higher number of CFUs was evident in fluconazole-resistant biofilms compared to those formed by susceptible isolates. ZnO-np reduced biofilm biomass and CFUs of dual isolate biofilms (P< 0.05). ZnO nanoparticles had a significantly (P< 0.05) greater effect on reducing fluconazole-resistant *C. albicans* biofilm biomass compared to susceptible isolates.

**Conclusion:**

Zno-np exhibits inhibitory effects on biofilms of both isolates. These findings provide an important advantage of ZnO that may be useful in the treatment of catheter-related urinary tract infection.

## INTRODUCTION

Urinary tract infection is the most commonly reported nosocomial infections worldwide. *C. albicans* is the most common cause of hospital acquired urinary tract infections (UTIs); however, there is a rapid change in the distribution of *Candida* species. Simultaneous increase of urinary candidiasis has led to the emergence of antifungal resistant *Candida* species (1).

UTIs are caused by *C. albicans* in 85%–95% of patients, and *C. glabra* and *C. tropicalis* are the cause of the disease in the rest of patients (2).

The pathogenesis of *Candida* species is related to certain factors such as the ability to escape host defense, adhesion and biofilm production, and the production of hydrolytic enzymes, such as proteases, phospholipases, and hemolysins (3).

Biofilms are independent and complex communities of organisms and can be considered as a strategy used by some microorganisms to protect themselves against the harmful effects of the host and the natural environment and to increase their chance of survival. *Candida* biofilms also bind to tissue and medical devices and cause problems by colonization and infection (4). Biofilm production also has a high level of antifungal resistance to microorganisms. In addition, the ability of the *Candida* species to produce drug-resistant biofilms is a major contributor to 80% of human infections (5).

Due to increased resistance to antimicrobial agents, infectious disease remains a public health problem worldwide. Hence, alternative strategies to treat fungal infections were sought and nanostructures were introduced as new antimicrobial agents. Although the exact mechanism of action of nanoparticles is not yet fully understood, it may be dependent on the following factors: composition, surface changes, inherent properties of nanoparticles, and nanoparticle concentration and species of fungi. Zinc oxide nanoparticle (ZnO-np) was the most toxic nanoparticle against *C. albicans* among the metal oxide nanoparticles. ZnO disrupts membrane integrity via production of reactive oxygen species that destroy fungi (6–10). In addition, production of hydrogen peroxide and Zn^2+^ has shown a key role in the antifungal activity of nanoparticles. ZnO nanoparticles have selective toxicity toward fungi, with minimal effects on human and animal cells (11).

The aim of this study was to determine the effect of ZnO-np solution in the surface catheter on *C. albicans* adhesion and biofilm formation.

## MATERIALS AND METHODS

### Sampling, culture, and identification of yeast.

A total of 260 urinary catheter of inpatients in Sayad Shirazi hospital in Gorgan, North of Iran, were used in this study. The Ethics Committee of Golestan University of Medical Sciences approved this study.

Sonication of the catheters, and scraping of the catheters using surgical blades followed by streaking of the blades on the agar plates, (C) scraping of the catheters followed by vortex mixing of the surgical blades, and (D) scraping of the catheters followed by sonication of the surgical blades. Microorganisms scrap of urinary catheter was cultured in SDA and their identification was performed with the use of common phenotypic and genotyping methods. Their antifungal sensitivity was confirmed by disk diffusion method. In this study, 113 susceptible isolates and 20 isolates resistant to fluconazole were determined.

### Preparation of ZnO nanoparticles.

To synthesize the ZnO nanoparticles, 5 grams of zinc acetate Zn (CH3COO) 2.2H2O (0.1 M) was prepared in 50 mLdistilled water under stirring. This solution was prepared using the sol-gel method in gelatin media and the samples were crystallized in single phase wurtzite structure. The size and morphology of ZnO NPs were measured using scanning electron microscope (SEM, Philps) (12, 13).

### Biofilm formation and drug treatment.

Isolates, including reference strains *C. albicans* ATCC 10231, 20 fluconazole-resistant *C. albicans* isolates, and 113 susceptible *C. albicans* isolates, were examined for biofilm formation in the presence of sub-MIC and MIC concentration of ZnO-np. Inoculate was prepared by collecting cells from overnight cultures in Yeast nitrogen base (YNB); then, they were washed and suspended in 0.85% saline to ~ 2 × 10^6^ cfu/mL. Doubling dilutions of ZnO-np were prepared in double-strength RPMI 1640 medium in 100 μL volumes in 96-well flat-bottomed polystyrene microtitre plates (Nunc, Roskilde, Denmark). Wells with no ZnO-np served as positive growth controls. Two dilution series were inoculated with 100 μL volumes of inoculum per isolate and resulted in final concentrations of ~ 1 × 10^6^ cfu/mL and 5–50 μg/mL ZnO. Each corresponding ZnO-np control well contained only ZnO solution and sterile growth medium. Microtitre plates were then incubated at 37°C for 24 hours. Then, the plates were inverted to remove well contents; the wells were washed 3 times with PBS to remove any residual planktonic cells. The extent of biofilm in each well was determined by Crystal violet dye, which was released by ethanol/acetone (80:20). Samples with optical density (OD_490_) of less than 0.1, 0.1–0.2, 0.2–0.3, and more than 0.3 were considered as none, weak, moderate, and strong biofilm former (14). These experiments were repeated at least 3 times.

### Adhesion to the catheter.

Seven isolates, including *C. albicans* ATCC 10231, 3 fluconazole-resistant *C. albicans* isolates, and 3 susceptible *C. albicans* isolates, were examined for adhesion to the catheter. Disks of catheter material (surface area, 0.5 cm^2^) were cut from catheters, sterilized with ethylene oxide (Caledonian Medical Ltd., Glasgow, United Kingdom), and placed in wells of 12-well Nunclon tissue culture plates. Standardized cell suspension (100 μL) was applied to the surface of each disk, and the dikcs were incubated for 2 and 48 hours at 37°C (adhesion period), with shaking at 75 RPM (15).

The catheter was analyzed by SEM to examine the adhered cells, the biofilm cell structure, and the effect of ZnO-np on biofilm prevention assay. Thus, after 2 and 48 hours of incubation, for both dual isolate experiments, the catheter was aspirated and the non-adherent cells were removed by washing the squares with 1 mL of PBS twice (0.1 M, pH 7). Also, MIC concentrations of ZnO nanoparticles the resistant and susceptible isolates of fluconazole 15 and 28 μg / ml respectively were added to the preformed biofilms and incubated at 37°C under agitation (120 rev/min) for an additional 24 hours. For controls, dual isolates biofilms were preformed on the catheter and incubated in RPMI without the addition of ZnO. The effect of ZnO on preformed biofilms was examined by CFUs, and SEM was determined as described previously. All assays were performed in triplicate and on 3 separate occasions.

### Scanning electron microscopy.

Disks catheter was dehydrated using an alcohol series (70% ethanol for 10 min, 95% ethanol for 10 min, 100% ethanol for 20 min) and air-dried for 20 minutes. The catheters were then maintained in a desiccator and mounted on to aluminum stubs prior to observation; sputter was coated with gold and observed using an S-360 scanning electron microscope (SEM, Philips) (16).

### Statistical analysis.

Data were analysed by t test in SPSS version 18. Significance level was set at P < 0.05.

## RESULTS

### Structural and morphological studies.

ZnO-np produced in this study was white and spherical. The XRD results revealed that ZnO has hexagonal wurtzite structure, and the peaks could be indexed according to JCPDS card, No.79−2205, with a = 0.3249 nm and c = 0.5205 nm (17). Furthermore, the diffraction peaks have broadened implying that the ZnO nanoparticles have nanocrystalline nature.

**Fig. 1.**
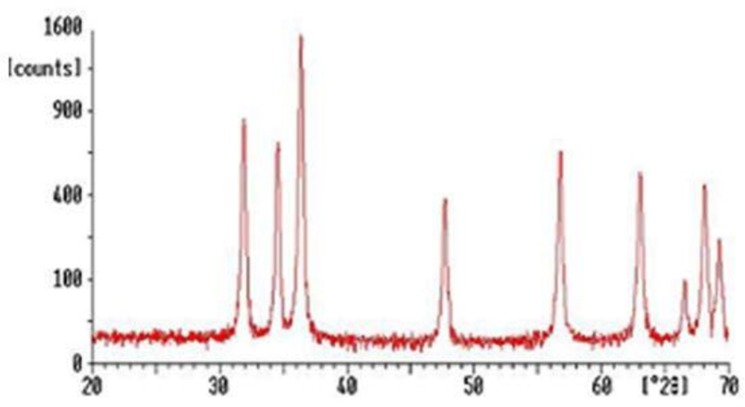
XRD image of ZnO nanoparticles

**Fig. 2.**
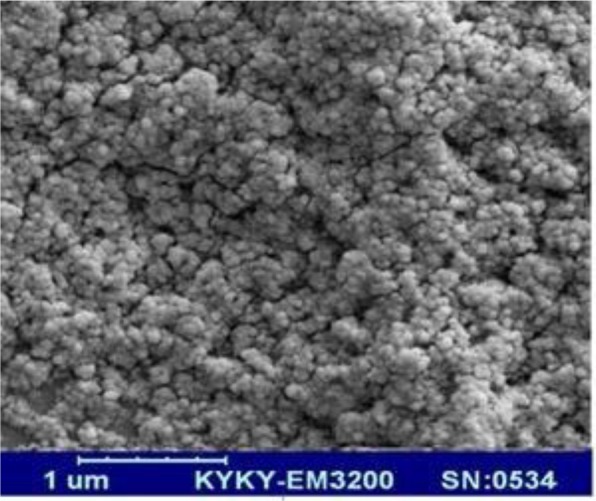
SEM image of ZnO nanoparticles

It is clear from the SEM image of ZnO that the particles are spherical with less agglomeration. The size of these spherical ZnO nanoparticles is in the range of 20–50 nm (Figs. 1 and 2).

### MIC of ZnO nanoparticles on *C. albicans* isolated from urinary catheter.

The MIC of ZnO–np on 113 (85%) susceptible isolates was 28±1.2 μg/mL and that of fluconazole-resistant isolates was 47± 2.8 μg/mL. The distribution of MIC value among properties' isolates was assessed and analyzed by statistical methods. Properties were as follow: the origin of fungi from inpatients, fluconazole-resistance, and history of urinary catheterization. The MIC of the ZnO nanoparticles of Fluconazole-resistant isolates was higher than the susceptible isolates (P<0.03).

### The effects of ZnO-np on biofilm formation in microplate method.

Most isolates from urinary catheter (60%) produced weak biofilms in SDB, containing 8% glucose (Ramage et al., 2001). ZnO-np at 29 μg/mL concentration fully inhibited biofilm formation in 90 (80%) of susceptible *C. albicnas* isolated from urinary catheter and 50 μg/mL concentration 20 (100%) in fluconazole-resistant isolates. In 20% of susceptible isolates the optical density of biofilm formation was reduced from moderate or strong levels to a weak level. In culture media, which contained the sub-MIC of ZnO-np, reduction in biofilm formation was observed in 58.33% of susceptible isolates and 60% of fluconazole-resistant isolates. (All isolates used in this experiment without the presence of ZnO nanoparticles, produced moderate or strong biofilms in the medium), None of the urinary catheter isolates of *C. albicans* showed a ful inhibition of biofilm formation at this concentration of ZnO-np. Our results revealed that the effect of ZnO-np is much greater on the fluconazole-resistant isolates and isolates with strong biofilms compared to susceptible isolates and weak biofilms (Tables 1 and 2).

**Table 1.** The effects of ZnO-np on the biofilm formation of susceptible *C. albicans* from urinary catheter isolates by crystal violate method.

**Biofilms**	**Before treatment with ZnO-np**	**After treatment with MIC of ZnO-np**		**After treatment with MIC of ZnO-np**		
		No biofilm	Weak	No biofilm	No biofilm	Weak
Weak	68^1^ (60%)^2^	48 (80%)	20 (20%)	-	48 (80%)	20 (20%)
Moderate	26 (23.3%)	21 (80%)	5 (20%)	-	21 (80%)	5 (20%)
Strong	19 (16.7%)	6 (33.33%)	13 (66.66%)	-	6 (33.33%)	13 (66.66%)
Total	113	117 (80%)	41 (20%)	-	117 (80%)	41 (20%)

1. Number of isolated used in table.

2. % isolated: % isolated used in table.

**Table 2.** The effects of ZnO-np on the biofilm formation of fluconazole-resistant *C. albicans* from urinary catheter isolates by crystal violate method

**Biofilms**	**Before treatment with ZnO-np**	**After treatment with MIC of ZnO-np**		**After treatment with sub-MIC of ZnO-np**		
		No biofilm	Weak	No biofilm	Weak	Moderate
Weak	-	-	-	-	-	-
Moderate	4^1^(20%)^2^	4 (100%)	-	-	2 (50%)	2 (50%)
Strong	16 (80%)	16 (100%)	-	-	10 (62.5%)	6 (37.5%)
Total	20	20 (100%)	-		12 (60%)	8 (40%)

1. Number of isolated used in table.

2. % isolated: % isolated used in table.

### SEM analysis of *Candida* adherence and biofilms on the catheter.

**Fig 3.**
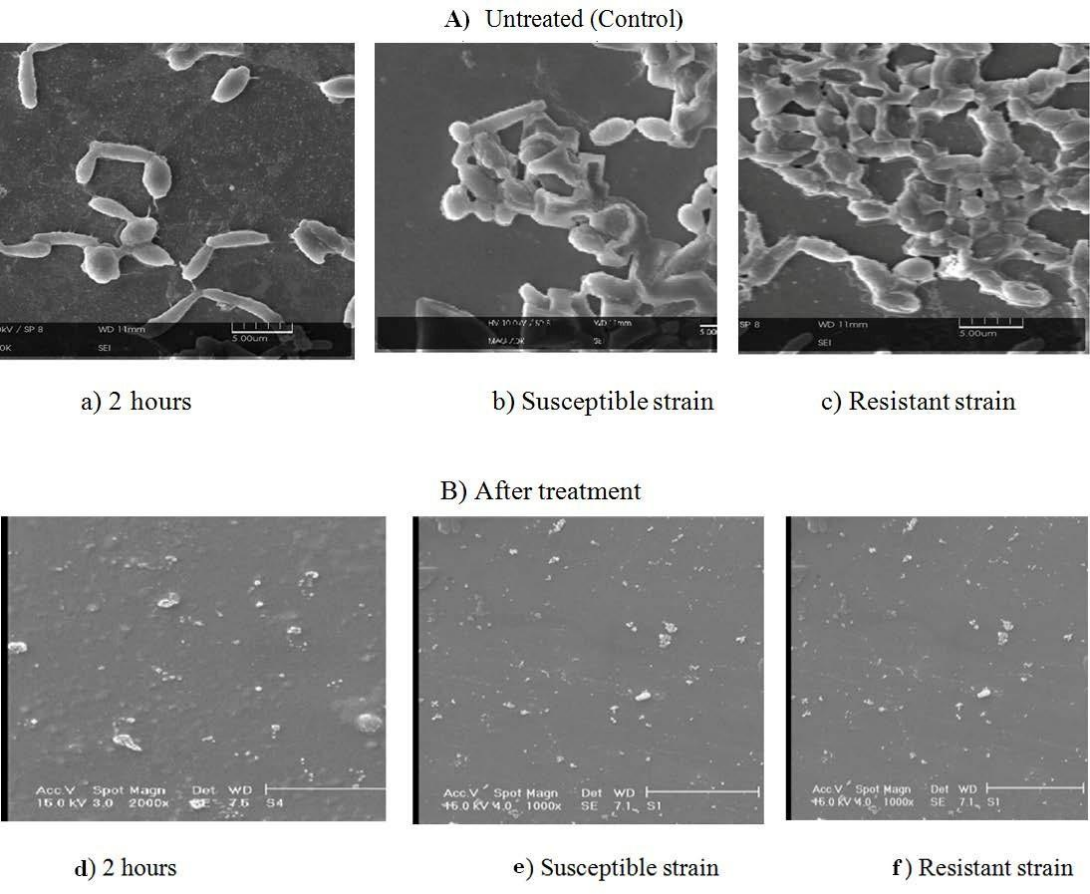
**A).** Scanning electron microscopy of A) Untreated (control; susceptible and Flu-resistant *C. albicans* isolates biofilm from urine on the catheter surface at 2 h (a) and 48 h incubation (b, c). Magnification-1000. B) Preformed biofilm of *C. albicans* treated with ZnO-np. A Preformed biofilm in 2 hours (d) and preformed biofilm after 24 h susceptible and Flu-resistant *C. albicans* isolates biofilm from urine on the catheter surface treatment of ZnO-np on the catheter surface (e, f). Cells were incubated for 48 h and treated with ZnO-nap was 68 and 100 μg/ml for 24 h. Pre-biofilm the treated was almost real without hyphae and clearly reduced in the number of cells, disruption cell wall is seen in the treated preformed biofilm.

**Fig. 4.**
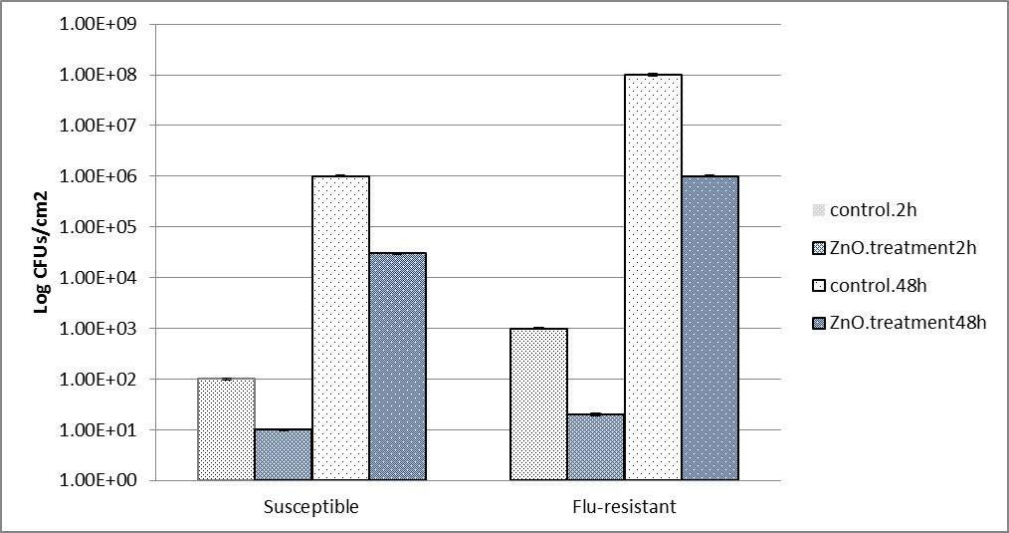
Mean absorbance (OD at 490 nm) and 95% confidence intervals obtained in the biofilm formation assay (48 h) for all experimental conditions evaluated adherence and biofilm formation of susceptible *C. albicans* and Flu-resistant *C. albicans* urinary isolates after colonization of the catheter. (A) Log 10 number of cells per unit area of the catheter; Effect of 72 μg/ml of ZnO nanoparticles for susceptible *C. albicans* and 100 μg/ml of ZnO nanoparticles for Flu-resistant *C. albicans* urinary isolates preformed biofilms of urinary isolates. The biofilms were analyzed after 24 h of treatment by determination of the number of cultivable cells. Results statistically different from the respective control (P <0.05); Statistically significant (P< 0.05) susceptible and Flu-resistant isolates colonization results. Error bars represent standard deviation. * Statistically significant (P< 0.05).

SEM was used to analyze the adherence and biofilm structure of *Candida* isolates biofilms (Fig. 2). Both susceptible *C. albican*s and fluconazole-resistant *C. albicans* isolates from rurinary catheter adhered to the surface catheter after a 2-hour incubation. Moreover, it was apparent that susceptible *C. albicans* and fluconazole-resistant *C. albicans* isolates from urinary catheter adhered similarly, without statistical differences between the species, even when used in dual isolates experiments (P< 0.05). Two different approaches were employed to assess biofilm formation: enumeration of CFUs (Fig. 4) and SEM (Fig. 3A).

Biofilms had a dense compact structure in the isolated from urinary catheter, forming a multilayer that covered the entire surface catheter. The biofilm structure of susceptible *C. albicans* was less compact, with a thick layer of aggregated cells partially covering the surface catheter (Fig. 3A).

The effect of ZnO-np completely inhibited the adhesion and forms of candidiasis urinary catheter isolates on the catheter compared to the control after 2 hours. More than 90% of the biofilms of isolated from urinary catheter were destructed after 24 hours of incubation with ZnO nanoparticles and small spherical cells (Fig. 3B).

The effect of ZnO-np against preformed biofilms was assessed based on the number of CFUs (Fig. 4).

The results (Fig. 4) showed that the ZnO nanoparticle can be an inhibitor of susceptible and fluconazole-resistant of *C. albicans* isolates on the catheter surfaces. To highlight this, there was a 15-fold reduction in susceptible *C. albicans* isolate CFUs biofilms treated with ZnO-np as compared to controls. An even greater (100-fold) reduction was evident when fluconazole-resistant *C. albicans* isolates biofilms were treated with ZnO-np that conforms to the results of Tables 1 and 2 (Fig. 4). In dual isolates, a reduction in the number of cells was again evident compared to controls (P <0.05).

## DISCUSSION

The zinc oxide nanostructure was successfully synthesized using the sol-gel method. The results showed that ZnO is spherical. The structure was successfully synthesized by the sol-gel method in the nanosize range of about 20–50 nm. Images from XRD and scanning electron microscope showed that ZnO-np good was made (Figs. 1 and 2).

It has been reported that the smaller the particles, the greater the antimicrobial effect because of the more surface area for interacting with microorganisms (8). Nanoparticles smaller than 10 nm are not only found on the surface of the cell membrane but also inside the fungi. Thus, the size of the ZnO-np synthesized in this study may have accounted for the activity of the prepared solution against *C. albicans.* The growth inhibition of *C. albicans* isolated from urinary catheter was complete in the presence of ZnO-np at a concentration of 100 μg/mL. The synthesized ZnO-np solution was also effective against sessile cells at the highest concentration evaluated.

The initial objective of this study was to compare the catheter colonization and biofilm formation by susceptible *C. albicans* and flu-resistant *C. albicans* isolated from urinary catheter. Results showed that both isolates adhered to the similar extent to the catheter (Fig. 3A). These findings are in agreement with those in previous studies examining *Candida* isolates adhesion on the same medical device surfaces (18). It is known that initial attachment of cells to a substratum is closely followed by cell division, proliferation, and biofilm development (19). The results of biofilm formation of *C. albicans* isolates were shown on the catheter surfaces, susceptible *C. albicans* biofilms yielded a lower total biomass compared to flu-resistant *C. albicans* isolated from urinary catheter (Fig. 3B) when CV staining was used as an indicator of biofilm formation. However, this direct comparison of biomass may be misleading as susceptible *C. albicans* cells are physically smaller than flu-resistant *C. albicans* isolated from urinary catheter (Fig. 3A) (21). Interestingly, susceptible *C. albicans* biofilms, despite having a lower biomass (Fig. 3B), contained higher number of cultivable cells compared to flu-resistant *C. albicans* isolated from urinary catheter biofilms (Fig. 3A).

Susceptible *C. albicans* exhibits significant differences with flu-resistant *C. albicans*, including relative cell size (as mentioned). In another context, Ez-Azizi et al. (22) showed the efficient adherence of flu-resistant *C. albicans* isolates to pre-formed susceptible *C. albicans* biofilms in a catheter model and also suggested the possible co-aggregation of these 2 isolates.

A biofilm environment is an important characteristic in promoting persistence of *Candida* in the host, as biofilms have a greater resistance to removal by host factors and are more tolerant to administered antifungal therapies (23, 24). *Candida* cells are indeed adept at forming biofilms on the catheter, as evident in this study (Fig. 3A). It is this property of biofilms that has led to the recognition of an urgent need to find new and alternative antimicrobial approaches that are effective against biofilms.

The fugicidal effects of ZnO-np treatments, such ZnO nanocomposites and ZnO, have recently been established (25). ZnO-np has also been reported to exhibit inhibitory effects on bacterial biofilm formation and candidal biofilm formation (26, 27). However, the antimicrobial activity of ZnO-np against preformed *Candida* biofilms has received comparatively little attention. Consequently, the present study evaluated the antimicrobial effects of ZnO-np on preformed dual isolates (flu-resistant *C. albicans* isolates and susceptible *C. albicans*) biofilms on the catheter. Previous investigations have identified the fungicidal effects of these 2 agents relative to the MICs of ZnO-np (5–50 μg/mL) against both isolates (28, 29). In the present study, experiments on preformed biofilms revealed that both ZnO-np significantly inhibited susceptible *C. albicans* and flu-resistant *C. albicans* isolates biofilms in terms of the number of cultivable cells (Fig. 3A) and total biomass (Fig. 3B). With regards to the number of CFUs recovered from urinary catheter isolates biofilms, it was evident that ZnO-np had a similar effect against susceptible *C. albicans* but was less effective against flu-resistant *C. albicans* isolates (Fig. 4). In this study, CV staining was used to quantify the total biofilm biomass since ZnO-np may also have had effects on the biofilm matrix. Results showed (Tables 1 and 2) that both agents caused a significant reduction in total biomass, with ZnO-np being more active against *C. albicans* from urinary catheter isolates biofilms. In the case of ZnO-np, it has been reported that interactions of the nanoparticles with microorganisms in biofilms induce particle aggregation (30, 31). Stewart and Franklin (32) reported that biofilm features, such as oxygen concentration, biofilm substrate, pH and biofilm composition, may influence this ZnO-np aggregation and consequently the diffusion and effect on biofilm cells. Such findings may be of significant importance because of the similar activity of ZnO-np against biofilms in these studies. The concentration and size of ZnO-np found to be effective in this study were lower than those previously reported by other authors to be toxic *in vitro* against human cells (33, 34).

## CONCLUSION

This study showed that susceptible *C. albicans* and flu-resistant *C. albicans* isolates urinary catheter were able to colonize the surfaces catheter in dual isolate biofilms. Flu-resistant *C. albicans* from urinary catheter biofilms yielded a higher total biomass compared to susceptible *C. albicans* isolates. This observation was expected as it reflects the frequently recorded incidences of urinary catheter candidiasis associated with the presence of these 2 *Candida* isolates. Additionally, it was shown that ZnO-np exhibited antifungal activity against both dual isolates preformed biofilms. Thus, given the inhibitory effects of ZnO-np against candidal biofilms seen in the present study, its use as an alternative treatment for urinary catheter candidiasis (particularly those involving the catheter biofilms) should be considered.
